# Writing up your clinical trial report for a scientific journal: the REPORT trial guide for effective and transparent research reporting without spin

**DOI:** 10.1136/bjsports-2021-105058

**Published:** 2022-02-22

**Authors:** Thomas Bandholm, Kristian Thorborg, Clare L Ardern, Robin Christensen, Marius Henriksen

**Affiliations:** 1 Department of Clinical Research, Copenhagen University Hospital, Amager and Hvidovre, Copenhagen, Denmark; 2 Department of Occupational and Physical Therapy, Physical Medicine & Rehabilitation Research – Copenhagen (PMR-C), Copenhagen University Hospital, Amager and Hvidovre, Copenhagen, Denmark; 3 Department of Orthopaedic Surgery, Copenhagen University Hospital, Amager and Hvidovre, Copenhagen, Denmark; 4 Department of Clinical Medicine, University of Copenhagen, Copenhagen, Denmark; 5 Department of Orthopaedic Surgery, Sports Orthopedic Research Center – Copenhagen (SORC-C), Amager-Hvidovre Hospital, Faculty of Health Sciences, Copenhagen University, Copenhagen, Denmark; 6 Musculoskeletal & Sports Injury Epidemiology Centre, Department of Health Promotion Science, Sophiahemmet University, Stockholm, Sweden; 7 Sport and Exercise Medicine Research Centre, La Trobe University, Melbourne, Victoria, Australia; 8 Department of Family Practice, University of British Columbia, Vancouver, British Columbia, Canada; 9 The Parker Institute, Section for Biostatistics and Evidence-Based Research, Copenhagen University Hospital Bispebjerg Frederiksberg, Copenhagen, Denmark; 10 Department of Clinical Research, Research Unit of Rheumatology, University of Southern Denmark, Odense University Hospital, Odense, Denmark

**Keywords:** methods, sports medicine, randomized controlled trial, research, education

## Abstract

The REPORT guide is a ‘How to’ guide to help you report your clinical research in an effective and transparent way. It is intended to supplement established first choice reporting tools, such as Consolidated Standards of Reporting Trials (CONSORT), by adding tacit knowledge (ie, learnt, informal or implicit knowledge) about reporting topics that we have struggled with as authors or see others struggle with as journal reviewers or editors. We focus on the randomised controlled trial, but the guide also applies to other study designs. Topics included in the REPORT guide cover reporting checklists, trial report structure, choice of title, writing style, trial registry and reporting consistency, spin or reporting bias, transparent data presentation (figures), open access considerations, data sharing and more. Preprint (open access): https://doi.org/10.31219/osf.io/qsxdz.

## Introduction

You worked hard as the primary investigator of a clinical research project. You spent months preparing the project,^1^ and perhaps years collecting and analysing data. You are now ready to report the work as a scientific paper (hereafter ‘trial report’), and submit it to a peer-reviewed, academic journal. You aim for quality and transparency because you want the end-user to be able to read-and-implement for clinical work or read-and-replicate for research. Your coauthors have different and contrasting input to your manuscript draft. How do you navigate this scenario?

Let us introduce the REPORT guide. It is intended to improve reporting of clinical research in general.^2^ It is not intended to replace established reporting checklists such as Consolidated Standards of Reporting Trials (CONSORT)^3^—they are always your ‘first choice’ reporting guidance resources. Rather, we intend the REPORT guide as a ‘How to’ implementation guide and directory that holds tacit knowledge (ie, learnt, informal or implicit knowledge) and references to sources of information about effective and transparent trial reporting. We have included information on topics we have struggled with ourselves as authors and see authors struggle with when we review or edit submitted clinical trial research. We published the PREPARE trial guide in 2017^1^ which aimed to assist in the preparation and planning of clinical trial research. The REPORT guide is a natural extension of PREPARE—focusing on reporting of clinical trial research. If you used the PREPARE trial guide^1^ to plan your research, the REPORT guide will likely help you report it. The REPORT guide can also function as a stand-alone guide to help you report research no matter how it was prepared.

The REPORT guide provides information to help improve reporting quality and transparency. The focus is the randomised controlled trial (RCT) (hereafter ‘trial’), but the guide is useful for other study designs.

## The CONSORT checklist and CONSORT-based web tool writing aid tool: an important first step

An important first reporting step is to locate a reporting checklist that matches your study design. A comprehensive list of reporting checklists can be found at the EQUATOR network’s website.^4^ For a trial, the appropriate reporting checklist is the CONSORT checklist^3^ for which there are several extensions that may be relevant. We encourage you to go to the ‘Toolkits’ section at the EQUATOR network’s website^5^ where you can find information to help you select the appropriate reporting checklist. You may also find the CONSORT-based WEB tool (COBWEB)^6^ useful in your writing and checklist adherence. As stated on the COBWEB(site): ‘COBWEB is an online manuscript writing aid tool intended to guide authors through the process of manuscript writing of RCTs in line with the Consolidated Standards of Reporting Trials (CONSORT) and its subsequent extensions’. We highly recommend you use this tool, as it will facilitate effective trial reporting. It will help you avoid many of the documented problems with CONSORT adherence, such as poor reporting of randomisation methods or description of sample size estimation.^7^

For more information on reporting checklists: 1 4 6 8 9

## Keep the trial protocol and registration next to you as you write the report

Journal reviewers and editors will be some of the first professional readers of your trial report when submitted to an academic journal. They want to know if you did what you set out to do and—if not—why you made changes. They will look at your trial registration and protocol, if publicly available or submitted with the trial report—comparing the information in the trial registry to that in the trial report and looking for consistency for important trial characteristics. Authors of systematic reviews will do the same when they include your trial—once published—in their review and assess bias, for example, in the selection of your reported results.^10^

**Figure 1:**
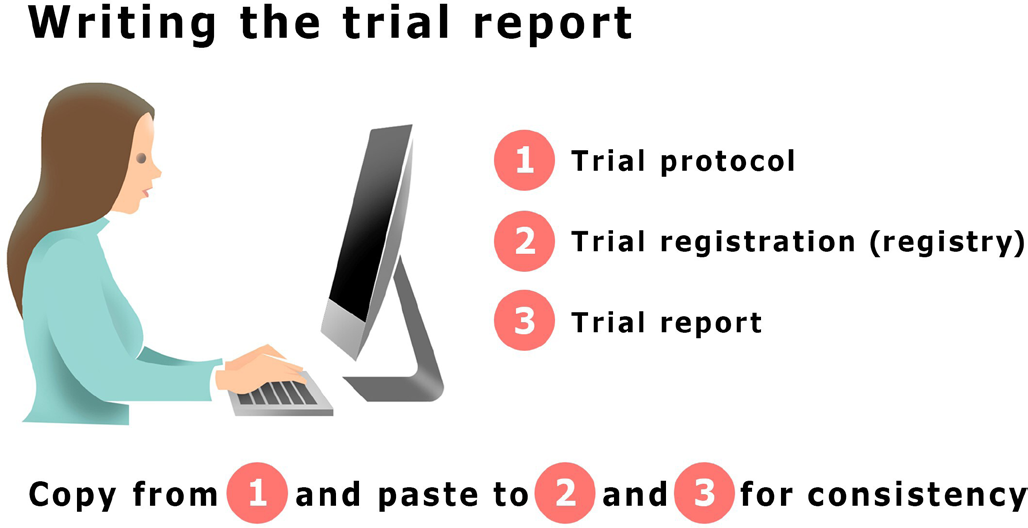
We encourage you to have the protocol open and trial registration available when you write. If you use a copy-paste approach, it will facilitate consistency between trial protocol, registration and report.

We encourage you to take the same approach as reviewers and editors when you write your trial report. Have the trial protocol open and the registration available when you write—generally use a copy-paste approach for important trial characteristics to increase transparency and consistency for two related work packages of the same research project (figure 1). Sometimes the trial report will be flagged by plagiarism checkers that journals use because the methods sections are very similar. Ensure you reference previous work and consider presenting the argument for this approach in the cover letter and/or the trial report itself. The guide ‘Avoiding Plagiarism, Self-plagiarism, and Other Questionable Writing Practices: A Guide to Ethical Writing’ by Dr Miguel Roig is a helpful and detailed resource.^11^ Roig made the case for more editorial flexibility when it comes to textual reuse of technical descriptions—especially for writers who do not have English as their first language.^12^ Finally, check any author/publisher copyright agreement if you have published your trial protocol to avoid any copyright infringement.

Using a copy-paste approach will help you report important trial items in the same order and with the same wording as used in the registration. Examples of important trial items include aim, selection criteria and outcomes. If you deviated from the plan (common and acceptable with a reasonable explanation) transparently report it and why. Many journals require that you upload the trial protocol as a supplemental file to the trial report. A copy-paste approach creates a strong link between these two documents and increases readability. If wording cannot be copied and pasted 100% for consistency, we suggest you check carefully if the meaning is still the same. For example, you may have come up with a better title after having revised the trial report many times, or you realised that the trial objective could have had better wording.

## Writing your trial report

### Structure: Introduction, Methods, Results and Discussion

Most scientific journals prefer a trial report style that follows the IMRaD-structure (ie, Introduction, Methods, Results and Discussion).^13^ You can find useful generic information on how to structure scientific papers from the PLOS collection.^14^ We provide additional information relevant to the different sections of a trial report below.

### Crafting a ‘tempting title’

Declarative and descriptive titles are the typical types of titles a reader is likely to encounter in the sports & exercise medicine field. A declarative title declares the key message (often a key result; eg,’ Meniscus or cartilage injury at the time of anterior cruciate ligament tear is associated with worse prognosis for patient-reported outcome 2–10 years after anterior cruciate ligament injury: a systematic review’).^15^ A descriptive title describes what the reader can expect to find in the trial report (often the type of study, the population or the outcome; eg’ Which treatment is most effective for patients with Achilles tendinopathy? A living systematic review with network meta-analysis of 29 RCTs’).^16^ When crafting a title for your trial report, consider whether you are aiming to engage the reader, inform the reader, or both, and if possible finalise the title with the design of your study.

Scientific writers can be creative without being frivolous, trivial or unscholarly/unscientific. A tempting title does not mean one has engaged in ‘spin’.^17^ We recommend aiming for a declarative title where possible—the title is your chance to share a powerful first impression with your reader—although, we recognise you do not always have a choice. Adopting the engaging: informative style^18^ offers a way to let your creativity flow, without straying too far from scholarly conventions and inviting the stroke of an obstinate editor’s correction pen. Here is one example:’ Running themselves into the ground? Incidence, prevalence, and impact of injury and illness in runners preparing for a half or full marathon’.^19^

Your tempting title might comprise two, or even three, parts: (1) the hook: perhaps a play on words or a metaphor, (2) the key message, where you declare why the reader will want to read on or what she will find if she reads on, and (3) a key distinguishing feature of your trial report: perhaps a characteristic of the population, the type of trial (eg, double-blind superiority trial) or time frame for data collection). To facilitate correct PubMed indexing and identification, the CONSORT group^3^ encourages authors to include the study design in the title (eg, ‘an RCT’).

### The stylish academic writer: three suggestions to help you capture and engage your reader’s attention

Scientific writing and creative writing are not polar opposites. Our statement in the last section on tempting titles bears repeating: scientific writers can be creative without being frivolous or unscholarly. Like with your title, we recognise you may not always have a choice about some aspects of style (eg, some journals require third person perspective and forbid using first person pronouns like *‘*we measured quadriceps strength using an isokinetic dynamometer.’).

#### Suggestion 1: use concrete language and banish passive sentences

Consider replacing ‘There are numerous approaches to the quantification of training load’ with ‘There are at least three tools to measure training load’. Even more concrete is: ‘We describe three tools clinicians could use to measure training load in recreational runners’ because (1) your reader knows how many ways to measure training load she can expect to read about, and (2) she knows something about the population. She also knows who is doing what to whom: clinicians (who) are measuring (doing what) the training load of recreational runners (whom). Numbered or ordered lists help you organise your thoughts and convey a clear message to your reader.

#### Suggestion 2: write in active sentences that are driven by active vivid verbs

Even when your writing context is constrained or less flexible (or perhaps inflexible) given journal requirements, we encourage you to address your reader directly—with active writing. One can choose to write concise, clear, coherent sentences or one can choose vague, passive, verbose sentences. Which sentence holds your attention as a reader? Concrete language uses active verbs (eg, describe, explore, compete, measure), avoids abstract nouns (eg, quantification, conversation, completion, effectiveness, discretisation) and clarifies who is doing what, to whom.

#### Suggestion 3: comb your manuscript for be-verbs and replace them with active verbs

Forms of be, including was, were, been, being, are, is or shown, are also juicy targets for writers who are aiming to resurrect their writing. Passive verb constructions like ‘can be measured’ or ‘were shown’ weigh your writing down. Try replacing a few *be-*verbs in each paragraph with active, vivid verbs (eg, masquerade, prescribe, roll, shun).

Writing well is a deliberate, careful and considered process. It is a craft that requires time and practice. You will find writing resources and suggested reading on renowned Professor of Linguistics and scientific writing coach Helen Sword’s website.^20^ Four of the five authors of the REPORT guide do not have English as their first language. In scientific writing, we use the three suggestions above. We also use a ‘how simple can you go’ approach to guard against major linguistic mistakes and to increase readability for readers whose first language is not English. In Lingard *et al*’s excellent Writing Craft series,^21^ they identify key grammatical challenges and offer practical tips for native Spanish, French, Dutch and German speakers who are writing in English (table 1).^22^

**Table 1:** Key grammatical challenges for Spanish, French, Dutch and German researchers writing in English

Language	Grammatical issue	Challenge	Tip
Spanish	Sentence structure	Your tendency may be to write longer sentences and use a variety of synonyms to avoid monotony	Try shorter sentences and word consistency as a strategy to improve clarity
	Prepositions	You may get confused trying to figure out, for instance, when to use ‘in’, ‘on’, ‘at’. In Spanish, you would only use the word ‘de’ for all those three	Spanish has significantly fewer prepositions. You may need to memorise the common English prepositions or use a search engine
French	Sentence structure	Even when you try to write simple and short sentences, it may seem to require more words to do so in French than in English	Avoid long convoluted sentences in English by seeking parsimony: check that all words are essential when critiquing your own writing
	Adjective positioning	You may be used to putting the adjective/qualifier after the noun/subject in French (eg, blue sky/ciel bleu), so your English writing sometimes does this	Revise each sentence by identifying the noun/subject and adjective/qualifier and verifying that the qualifier precedes the noun as per English word order convention
Dutch	Sentence structure	You may struggle with the position of adjuncts, what a sentence can ‘carry’ in subject position, and the limited freedom in ordering the elements of an English sentence	Avoid ‘heavy’ subject clauses (lots of information in subject position) and make sure the subject position houses the most important information in the sentence. Don’t fling around the parts of the sentence—that can create chaos, rather than cleverness
	Parallel structure	You may tend to use synonyms and variety in sentence structures to ‘polish’ your text. However, variety can compromise clarity and dilute parallelism	Put clarity before variety: avoid synonyms when possible. Try using parallel structure to strengthen your key messages
German	Sentence structure	You may be accustomed to writing longer, more complex sentences that try to build up tension	Aim for short sentences, put the main information first and avoid too many conjunctions
	Paragraphing	Your German paragraphs are supposed to combine several strands of thought, so the principle of paragraph unity can feel foreign	Focus on unity—one idea per paragraph. Start with a topic sentence that clearly signals that idea

The table is reproduced from Lingard *et al.* 21 https://link.springer.com/article/10.1007/s40037-021-00689-2%23rightslink under the terms of the Creative Commons CC BY 4.0 license https://creativecommons.org/licenses/by/4.0/. No changes were made.

Three things to try:

- Watch Professor Sword explain how to avoid nominalisations.^23^
- Run your writing through an online workout.^24^
- Train with some Wordcraft Workouts.^25^

### Abstract: the CONSORT-way

The trial report abstract is important because it will likely have many more reads than the full trial report. Most journals have a word limit for abstracts, and some have mandatory structure and headings. Each of these restrictions can pose a challenge when writing a clear, transparent and detailed abstract—you need to make every word count.^21^ If journal formatting allows, use the CONSORT for reporting randomised trials in journal and conference abstracts.^26^ It comes with an explanation and elaboration paper^27^ as well as an abstract item checklist, which can be downloaded from the CONSORT website.^28^ Preliminary work from the CONSORT group showed that all the checklist items can fit within 250–300 words.^27^ The CONSORT website also has a sample study that implements the CONSORT checklist.^29^ The sample study includes an example of how to write abstract results, which can be problematic.^30^

**Figure 2:**
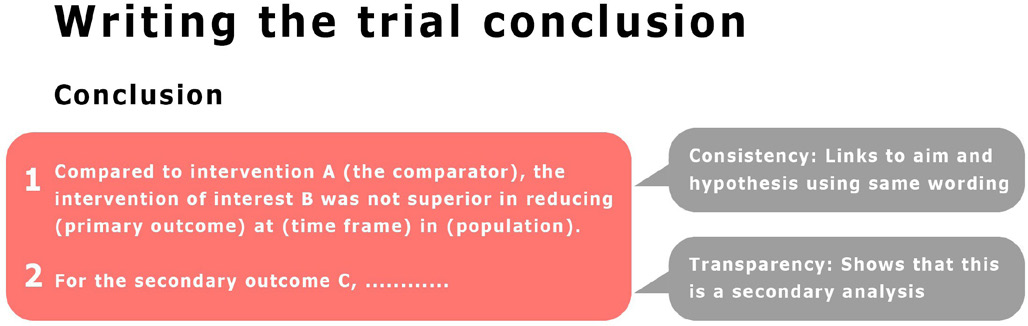
We encourage you to create a strong link between the conclusion and the trial aim and hypothesis if you think ‘aim’, ‘hypothesis’ and ‘trial design’ when you write the first line of the conclusion.

When you state the trial framework, for example, ‘superiority trial’ it creates an excellent link to what follows in the abstract. It links to which intervention the authors hypothesise to be superior to the comparator (objective); the main outcome and time frame that this is assessed (primary outcome and endpoint); indication of risk of bias (randomised vs analysed, blinding, trial registration), indication of superiority (effect size, between-group difference in response for the primary outcome) and claim of superiority as hypothesised (conclusion). To avoid unintentional reporting^31^ or spin^32^ biases in the conclusion, we suggest you reserve the first line to conclude on your objective and corresponding primary outcome and use PICOT^33^ as the framework (Population, Intervention, Comparator, Outcome, Time frame). For example, ‘Compared with intervention C (the comparator), the intervention of interest I was not superior in reducing O (primary outcome) at T (time frame) in P (population). We encourage you to then continue with secondary outcomes: ‘For the secondary outcomes X, Y and Z, we found that (…)’ (figure 2).

If your trial was prospectively registered, we suggest you state this at the end^26^ of the abstract as ‘Trial identifier: (number) followed by ‘(prospectively registered)’. If for some reason your trial was not registered before the first participant was included (prospectively/preregistered), we suggest you transparently report this at the bottom of the abstract as ‘Trial identifier: (number)’ followed by ‘(retrospectively registered)’. This is currently the editorial policy for all BMC journals when they consider retrospectively registered trials for publication.^34^ In the main trial report we encourage you to explain the reason for this practice and state if important trial changes occurred after the trial began, as there will be no publicly available record of your research intentions. If you posted your trial report as an open access preprint, we encourage you to add the preprint information to the bottom of the abstract. In the REPORT guide, you will find an example of this use (more info about preprints below). It will help the reader find an open access version of your trial report and link the two documents via the digital object identifier (DOI)^35^; ‘Preprint (open access): http://doi.org/(doi number)’. We suggest you use a copy-paste approach for important abstract information, so that it is consistent with the trial registration and/or published protocol as well as main trial report document (eg, aim and conclusion).

For more information on abstract reporting: 21 27 36

### Introduction: the ‘why’ of your trial

In this section, we encourage you to present the ‘why’, that is, an argument for why your trial is needed. If the ‘why’ is not clear to you and your coauthors, it will be difficult to convey it in a trial report. Readers are already motivated because they screened your title and abstract for relevance and results. However, the Introduction helps the journal’s reviewers and editors judge the importance of your trial report. It is therefore essential to making it interesting, while at the same time concise and describe the knowledge gap that your trial is intended to fill.

Your introduction should present the scientific background and rationale.^3^ It should follow the background section of your protocol, as the reason for doing the trial has not changed. Thus, the introduction can more or less be copy-pasted from your protocol. During the planning and conduct of your trial, however, others may have published relevant research findings. They may support or oppose your results but should be mentioned. The introduction should include a summary of relevant studies as an up-to-date systematic review or at least include the latest published systematic review on your topic. It is important not to be selective in this literature review as it may mislead the reader, increase the risk of confirmation bias^37^ or unintentionally communicate that the knowledge gap is larger or more important than it is. Consider letting the reader know that you have made steps to avoid this by stating that you have scrutinised all available evidence and use the best available evidence to inform the need of the trial: ‘The latest systematic review with meta-analysis on the effectiveness of (your intervention) on (your outcome of interest) concluded that (main finding). This is supported by two recent trial reports published after the systematic review by (author)’.

While the background information in a trial protocol oftentimes is very lengthy, the introduction part of a trial report can be shorter. Consider who will read your trial report and try to direct the introduction to that audience. For specialty journals it is not necessary to state general knowledge in the field. If you write about treatment of sports injuries and intend to publish in a sports medicine journal, it is unnecessary to write elaborately about the prevalence, costs, injury mechanisms or importance of treatment effectiveness. Readers of the journal will know this information already. Focus more on your trial rationale, specific research question, and aim. By cutting to the chase, you will save words that are better spent elsewhere.

End the introduction by stating the aim or the objective of your trial and include the hypothesis. Aims and hypotheses are not always easy to differentiate, but hypotheses are typically more specific and relate closely to the chosen trial design, outcome measures and statistical analysis plan (SAP). This is the most important part of the introduction. We suggest you copy/paste from the trial registry and/or published protocol for consistency (figure 1). We also suggest using the copy/paste approach for abstract and main trial report so that the aim in the abstract is the same as the one in the main trial report.

For more information on systematic reviews to fully use previous research: 38 39

### Methods: the ‘how’ of your trial

In this section, we encourage you to present the ‘how’ of your trial. What did you do in order to answer the ‘why’? The methods section is a detailed description of what was done and serves at least two main purposes: (1) to provide enough information to allow the reader to critically appraise and interpret the results, and (2) to convey as many details as possible so other researchers (in principle) will be able to replicate you trial entirely or in part. For clinical application of your trial results, it is important to give detailed descriptions of the population selection, assessment methods, and interventions. Other important aspects of the methods section are central for evaluating the scientific quality, validity and reliability of the trial.

Ideally, the methods section should be a replica of your trial protocol. But completing a trial without ‘bending the rules’ laid out in the protocol is practically impossible. It is therefore important to report any deviations and violations of the protocol. It is not a ‘scientific crime’ to deviate from the protocol, but it is important to report any deviation with potential bearing on your primary and important secondary outcomes (and thus on the interpretation of the entire trial). It is particularly important to declare ‘planned’ deviations, such as changes to eligibility criteria (eg, due to safety or slow recruitment), changes in instruments (eg, change of MRI-scanner due to breakdown). We suggest you report the deviations with reasons and describe what you did.

When you write your trial methods, imagine that your trial report 1 day will be scrutinised as part of a systematic review or clinical guideline. Reviewers will appraise your trial report on the lookout for flaws (or risks of bias). While you may have conducted your trial scrupulously (ie, with a low risk of bias), reporting can be incomplete. Reviewers may be uncertain about aspects of your trial methodology, which may mean they must downgrade the quality of your trial. We suggest you consult The Cochrane Handbook for Systematic Reviews of Interventions.^40^ It provides detailed information on how to appraise individual trials, and provides you useful hints on what reviewers are looking for. For example, a reviewer may look for the phrase ‘sequentially numbered, opaque, sealed envelopes’ when assessing risk of bias for ‘Allocation concealment’. Knowing this, will help you clearly report how this was done in your trial.

For more information on how to report protocol deviations and risk of bias assessment: 40 41

### Methods: outcomes

The CONSORT checklist items for ‘Outcomes’ ask you to report ‘Completely defined pre-specified primary and secondary outcome measures, including how and when they were assessed’ and ‘Any changes to trial outcomes after the trial commenced, with reasons’.^3^ If you use a copy-paste approach, it will be easy to copy from your protocol and preregistered trial summary and paste into the trial report. It will create consistency with regards to, for example, number of outcomes, outcome hierarchy and wording. If for some reason you had to add or remove outcomes during the trial, we recommend you report this transparently, with reasons.

The COMPare trials project^42^ team systematically checked every trial published in the top five medical journals between October 2015 and January 2016, with the purpose of searching for misreported findings and outcome switching. This team’s effort revealed a large degree of inconsistency in outcome reporting.^43 44^ If for some reason, you had to make changes to your trial outcomes after the trial began, state this transparently and give reasons for the changes. If you plan to report collected outcomes in subsequent (secondary) trial reports, we suggest you state in the primary trial report that the outcomes were collected—consistent with the trial registry—and that you plan to report them in a subsequent report. This could be the case if you collected mechanistic and more exploratory outcomes in your trial, such a blood samples that await future advanced molecular analysis. Stating that these were collected will help you avoid misreporting of outcomes. The COMPare trials project^42^ state in their Frequently Asked Questions-section: *‘Question: What if some outcomes are reported in a different publication? Answer: This is fine, as long as this fact has been declared in the trial publication. For example, if a trial says here we are reporting A B and C, in a subsequent paper we will report X Y Z then the outcomes X Y Z are not considered as unreported, and they are removed from the denominator.*’.^45^

For more information on how to report trial outcomes: 3 42 46

### Methods: interventions

Proper reporting of interventions is especially important for clinical application of your trial interventions, correct interpretation of your trial results, comparison with other trials (with similar interventions) and ability to inform new research questions. Unfortunately, intervention reporting is generally poor.^47^ To help you report your interventions, The Better Reporting of Interventions: Template for Intervention Description and Replication checklist was developed.^48^ It will help you make a complete and thorough generic description of the interventions. You may also need to consult a more intervention-specific guide or reporting checklist, such as the Consensus on Exercise Reporting Template for exercise trials.^49^ We suggest you describe any ‘usual care’ or other comparator intervention using the same standards and checklists. For some comparators, reporting checklists have been developed—one was just developed for placebo and sham controls.^50^ You may already have published a detailed description of your intervention and comparator as part of a published protocol. In case changes were made to the intervention or its delivery during the trial, consider if the description needs to be updated and submitted with the trial report as supplemental material. It will help both replication and clinical implementation of your trial results. In case you have not already published a detailed intervention description, consider publishing it as supplemental material to your trial report. It will help you if the journal has a word limit for the main document.

For more information on how to report trial interventions: 48 49

### Methods: sample size

This sample size paragraph is intended to outline how you, in the trial planning phase, ensured that the trial would have the required statistical power to identify whether a difference of a particular magnitude (the target difference) exists for the primary outcome. It is also intended to show that you did not include any extra participants than were needed for the trial. As you did all the thinking already, it should be feasible to copy/paste from the trial protocol. The basics of calculating sample size are covered in substantial detail in the PREPARE Trial guide.^1^

For more information on how to determine and report the target difference and sample size estimation for a trial: 51

### Methods: statistical analyses

The problem of poor statistical reporting is long-standing, widespread, potentially serious and yet is largely unsuspected by many readers of the biomedical literature.^52^ General guidance on how to write SAPs is now available^53^ and provides recommendations for a minimum set of items that should be addressed for clinical trials before analysing data.^53^ If you have not written a specific SAP as part of the trial protocol,^1^ we recommend that you consult a biostatistician and write one before viewing any data or starting the analysis.

An SAP ensures that the statistical methods are reported in sufficient detail to enable a knowledgeable reader (with potential access to the original data) to assess the appropriateness of the chosen statistical methods and the underlying assumptions, and to verify your reported results. In a SAP, the statistical methods are often described in great detail, and a complete copy-paste approach may be too much (given that most journals have restrictions on manuscript length). We therefore recommend always submitting the SAP (with final date on cover page) as supplemental material so that editors, peer reviewers and other readers can take a deeper dive into the statistical methods.

In the main text, we encourage you to give an extract of the primary statistical analyses (from the SAP). If you have stated a clear objective, the reader will be able to understand the primary purpose of the trial and what to expect to see next. We recommend that you describe fully the main methods for analysing the primary and key secondary objectives of the trial. It is common to analyse the data set under different assumptions—sensitivity analyses—to assess the robustness of the primary analyses. These are typically based on different strategies for handling missing data or analyses of different trial populations (eg, the per protocol population which is potentially biased but still informative). We recommend you carefully describe these strategies. Excellent educational resources exist to assist you. They include the CONSORT explanation and elaboration paper,^3^ the SAMPL guidelines for statistical reporting,^54^ and the recently developed Checklist for statistical Assessment of Medical Papers statement.^55^

For more information on how to report statistical analyses for a trial: 3 54 55

### Results: attrition

Attrition can introduce bias in your trial results if the characteristics of participants who are lost to follow-up differ between the randomised groups—especially if the differing characteristic is related to trial outcome measures.^56^ If you use the CONSORT flow diagram to illustrate the trial profile, we suggest you report the demographics of the participants included in the intention-to-treat population with descriptive statistics for each group. We encourage you to create an overview by preparing a classic table 1 of baseline characteristics using the outline from the CONSORT explanation and elaboration paper.^3^ You may also find it useful to supplement with table items as suggested by Dumville *et al* 56 and attach as a baseline appendix.

Reviewers will sometimes ask for results of statistical testing for baseline differences. The recommendation from the CONSORT group is clear: ‘Such hypothesis testing is superfluous and can mislead investigators and their readers. Rather, comparisons at baseline should be based on consideration of the prognostic strength of the variables measured and the size of any chance imbalances that have occurred’.^3^ It means you should subjectively judge if any differences between groups that will occur by chance due to randomisation is of a magnitude that you think is clinically relevant.

For more information on how to report attrition: 56

### Results: focus on the main analysis and between-group differences

Correct reporting of the results of the statistical analyses includes explicit estimates presented with appropriate indicators of measurement error or uncertainty, such as 95% CIs. Randomised trials are designed to analyse differences between groups, and the results should focus on these—not on changes within groups. However, it is helpful for transparent reporting and interpretation to present the estimates in each group. We strongly recommend that you avoid reporting only statistical hypothesis testing (eg, such as P values), as they do not contain much information and do not convey important information about effect sizes or precision of estimates. When you report p values, we recommend you report actual p values, rather than p<0.05, unless the value is very small (eg, p<0.0001).

We suggest you report your primary analyses first and hierarchically (primary outcome before secondary and other outcomes). This will most likely follow the hierarchy outlined in your trial protocol and SAP. By being consistent and use a copy/paste-approach, you will help the reader assess if you followed your SAP. We recommend you avoid interpretations or interpretative language in the results section, but instead help the reader by providing a direction of the results and whether it favours one of the groups. In cases where discrepancies between analysis sets occur among the primary analyses and the sensitivity analyses, we suggest you highlight them in the text. You may also need to devote more attention to interpreting the collective results because the confidence in the individual analyses is reduced.

During your data analyses, new and exciting ideas may arise, as well as unexpected findings that you did not consider during planning. Such results can be important and foster significant scientific advances. However, consider that your trial design may not support confirmatory analyses or statements of such findings, and it is important to state (both in the Statistical methods section and in the Results section) that these were not prespecified. Related to this topic is the situation where a peer reviewer asks for additional analyses of your data set; that is, frequently referred to as the ‘peer review pressure test’. These are often valuable and reasonable requests, but should very rarely replace the original analytical strategies, unless there are fundamental flaws in the trial design and/or the chosen analyses do not reflect the experimental design. We suggest that post hoc analyses requested by peer reviewers are reported in supplemental files and included in a response letter to the reviewers when submitting a revised manuscript.

For more information on how to report the results of statistical analyses for a trial: 54 55

### Results: transparent illustration of your data

Your tables and figures should ideally be able to stand alone (eg, in presentations and lectures). It is valuable to provide brief summaries of the statistical methods used (eg, as foot notes to tables and figures). The CONSORT checklist and explanatory paper have great examples and descriptions of how to make certain illustrations. On the CONSORT website, you will find a flow diagram template freely available for download.^28^ We suggest you add some additional information to the flow diagram, including numbers of participants included in the different analyses (eg, intention-to-treat and/or available case analysis) and number of imputations made for missing data, if applicable. For an example, please see Lysdal *et al* 57

Include specific information on your sampling strategy at the top of the flow diagram because it will facilitate interpretation of the trial findings with regards to clinical relevance. Consider reporting the total number of potentially eligible participants during the trial recruitment period and how many of these were assessed for eligibility, instead of only reporting the number of individuals assessed for eligibility. It allows the reader to judge how well the trial population represents all patients seen at the recruitment site while the trial was running. Because this issue relates to external validity it is important—but it is especially important if the trial findings have major implications for current clinical practice. Please see Clausen *et al* 58 for an example of how the number of potentially eligible participants can be incorporated into the trial flow chart. Please also see the rapid responses^59^ to the FIMPACT^60^ trial for a discussion of the importance of including the number of potentially eligible participants when trial findings have great implications for clinical practice.

**Figure 3:**
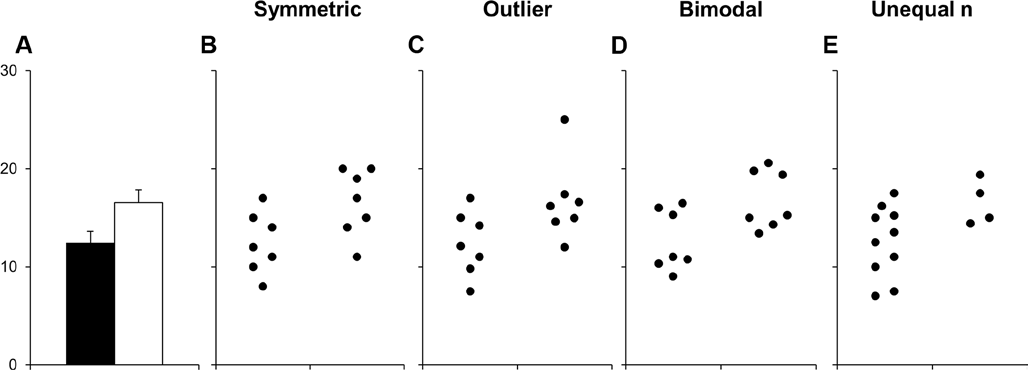
Many different datasets can produce the same bar graph. The figure and legend are modified from Weissgerber *et al* 61 https://doi.org/10.1371/journal.pbio.1002128 under the terms of the creative commons CC by 4.0 license https://creativecommonsorg/licenses/by/40/.

Results may sometimes merit a figure in the form of a graph. Many bar or lines graphs—based on continuous data with different distributions—can lead to the same bar or line graph (figure 3).^61^ Unless you include raw data in the graph, most information will be invisible to the reader. We encourage you to making it visible by using scatter plots instead of bar charts.

For more information on how to effectively use tables and figures in scientific papers:^61–63^

### Results: harms

When new healthcare interventions are studied, researchers tend to focus more on efficacy than safety. There is poor reporting of harms in trial reports across many clinical areas,^64^ which makes it difficult to obtain a true estimate of the benefit-harms ratio. The CONSORT extension for harms^65^ was developed to improve reporting of harms-related data in trials. Because the main focus of the CONSORT checklist is efficacy reporting, we suggest you supplement your trial reporting with the CONSORT extension for harms^65^ to improve reporting of harms-related data.

For more information on reporting of harms-related data: 65

### Discussion: consider clinical relevance and confirmation bias

The CONSORT checklist^46^ holds the overall framework for the discussion and items you should address, but scientific journals may have additional requirements. We suggest you use the CONSORT checklist to structure the discussion, and supplement with requirements from your target journal, if needed. We would like to highlight two important items: clinical relevance and confirmation bias.

We suggest you focus on the primary analysis and outcome. Your trial was designed first and foremost to provide a reliable answer in terms of the hypothesis for this analysis and outcome. The test statistics will determine if the difference between groups is statistically significant. Judging and discussing whether a statistically significant difference between groups is also clinically relevant should be easy at this point. You will already have argued in your trial protocol and sample size paragraph what minimum theoretical difference between groups you consider clinically relevant and why. Now that you have the observed difference between groups, the main issue is to compare the two and discuss the size of the observed effect. An important aspect of this discussion is the precision of the observed effect. In general, the larger the sample size of your trial, the greater the precision of the observed effect. The precision is reflected in the 95% CI of the observed effect. The greater the precision, the smaller the 95% CI and vice versa. We suggest a balanced discussion of the clinical relevance of the observed effect to include both its size (in relation to the predefined minimal clinically importance difference) as well its precision. It will help you avoid unintentional confirmation bias (please see below).

Biases come in many forms and can affect healthcare in many ways. There may be biases that you want to acknowledge specifically under ‘Limitations’ in the discussion because you think they may have influenced trial procedures or outcomes. We suggest you consider your own ‘confirmation bias’ when writing the discussion—or the whole trial report for that matter. As stated by the Catalogue of Bias Collaboration^37^: ‘Confirmation bias occurs when an individual looks for and uses the information to support their own ideas or beliefs. It also means that information not supporting their ideas or beliefs is disregarded.’ Being researchers, we argue that most of us unintentionally wish for our intervention to be superior to the comparator for several reasons: (1) we want to help patients by advancing the field, or (2) we think it will bring promotion or other academic reward. By being intentionally aware of our own confirmation bias, we can better stay clear of issues such as unintentional secondary analysis emphasis (spin) and selective referencing of work that support our own findings.

For more information on statistical significance, clinical relevance, spin and confirmation bias: 17 37 66

### Conclusion: what was your trial designed to test first and foremost?

When you write the trial report conclusion, we encourage you to think ‘aim’, ‘hypothesis’ and ‘trial design’. What was your trial designed to test primarily and how was this formulated in the aim? Was it to assess if the intervention of interest was better than (superiority trial), no worse than (non-inferiority trial), or whether it was as equally effective as (equivalence trial) the comparator? Using this line of thinking will help create a strong connection between aim, hypothesis and conclusion. It will also help you conclude only what the trial data support. If the aim of a superiority trial was ‘To investigate if I (Intervention of interest) is superior to C (comparator) in improving O (primary outcome) at T (timepoint) in P (population) and there was no difference in response between groups, the conclusion could start with: ‘Compared with C (comparator), I (Intervention of interest) was not superior in reducing O (primary outcome) at T (time point) in P (population). A very common mistake is to interpret the absence of evidence of superiority as evidence of equivalence or non-inferiority and conclude that the intervention of interest and comparator were equally effective or no different (for more details, please see refs 1 67).

Having addressed the main hypothesis, analysis and outcome the trial was designed to assess, we encourage you to proceed with interesting secondary analyses and—at the same time—inform the reader about the increase in risk of bias for these analyses: ‘For the secondary outcomes, X, Y and Z, we found that (………).’. When you conclude first on the primary analysis, you minimise the risk of unintentional reporting^31^ or spin^32^ biases. If your trial was more exploratory than confirmatory^1^—or had a flat outcome hierarchy with no single primary outcome—you may want to consider finishing the conclusion by acknowledging this. For example, ‘This finding needs replication in future trials’. Readers will often be interested in your thoughts on the implications of your trial findings. Some journals allow implication statements and others do not. If you do write about implications, we suggest you make it clear that this part of the conclusion is you speculating and conveying your expert opinion with phrasing like: ‘These findings may have implications for (……) insofar as (……).’. When you have finished writing the conclusion, check that it matches the trial aim and conclusion in the abstract.

## Sharing research data

Depending on national legislation, you may or may not be able to share the raw trial data. Data sharing is one way of increasing transparency and maximising the trial participants’ research contribution by making the data they provided broadly available for secondary research purposes.^68 69^ Data sharing is also expected by some non-private funding bodies.^70^ If you can share your trial data, there are some things that you may want to consider. They include practical steps to data management, anonymisation and storage.

For more information on data sharing: 71–74

## Alternative avenues for disseminating your research

### Preprint

When you are ready to submit your trial report to a scientific journal, consider publishing a preprint. A preprint is scientific work that has not undergone peer review and is not published in a scientific journal.^75^ It is typically a manuscript draft that is ready to be submitted to a journal for peer review. A preprint can also be an earlier manuscript version that you want to make public. One advantage of publishing a preprint is that it is assigned a DOI,^35^ which makes it searchable. Most publishers allow preprints,^76^ but we suggest you check the preprint policy of the scientific journal that you aim to submit your trial report to. Elsevier states: ‘Preprint: Authors can share their preprint anywhere at any time. If accepted for publication, we encourage authors to link from the preprint to their formal publication via its Digital Object Identifier (DOI). Millions of researchers have access to the formal publications on ScienceDirect, and so links will help your users to find, access, cite, and use the best available version. Authors can update their preprints on arXiv or RePEc with their accepted manuscript. Please note: Some society-owned titles and journals that operate double-blind peer review have different preprint policies.’.^77^

Submission to preprint servers is typically free and it creates an open access option, even if you end up publishing your trial report behind a paywall.^78^ It allows you to have crowdsourced feedback and to promote your open access research early (eg, during the period of peer review). Based on feedback, you can update your preprint version when you revise your manuscript. Some (but not all) publishers even allow you to update your preprint to the accepted (non-type set) manuscript version with proper reference to the journal publication. Please check the publisher’s preprint policy for guidance. If you look at the bottom of the abstract of this guide, you will see a link to an open access preprint. Had this guide not been published open access, an interested reader could see from the PubMed abstract where to find an open access full text (preprint).

For more information on preprints: 75

### Media

Researchers are familiar with social media platforms like Twitter for sharing new scientific content. When posting to social media, make space to include (1) the DOI^35^ and (2) an image—two simple steps to help make your post visible to attention metrics-aggregators like Altmetric and capture the viewer who might otherwise scroll past your post. Across research areas, the Altmetric score has been associated with number of citations, journal impact factor, press releases and open access status.^79 80^

Have you considered other forms of media? Researchers who embrace the rich ecosystem of digital media might find themselves partnering with clever infographics designers or using free (or freemium) websites to design their own. Consider writing for trusted outlets like The Conversation^81^—a news organisation that is dedicated to sharing information from the academic and research community, direct to the public, with ‘academic rigour and journalistic flair’. Sports medicine and sports physiotherapy journals including British Journal of Sports Medicine and Journal of Orthopaedic & Sports Physical Therapy have blogs dedicated to reaching a non-academic audience of clinicians, patients, athletes and coaches.

Consider approaching your academic institution’s media and communications department or press office. The staff are typically pleased to work with you to shape a press release, distribute the press release to mainstream media services, and connect with media contacts. Media and communications departments also share helpful tips for making your research visible to the media.^82^

## After publishing the trial report

After your trial report is published, consider (1) Is the ‘Trial status’ up to date in the trial registry? (2) Do I need to update the trial registry with a link to the published trial report and/or raw data if shared? (3) Do I need to report to funding bodies on the accomplished milestone (publication)? (4) Do I have a plan for disseminating the trial results other than the primary trial report? (5) Do I have a plan for storing and filing essential trial documents and data that adheres to national guidelines?

## Summary

We hope the REPORT guide is helpful and a valuable supplement to ‘first choice’ trial reporting tools, such as CONSORT. We aimed to incorporate tacit knowledge about reporting, and flag issues we have struggled with. Quality decisions in healthcare depend on reliable evidence of treatment effects. Good research reporting practice does not cure ‘diseases’ that arise from poor research methodology—it helps the reader see the illness and appraise the research quality. No research is perfect. We do not profess to produce and report perfect research that is free from ‘disease’ 100% of the time. We implore all researchers to commit to conducting (and reporting) clear and transparent research.

**What is already known**

- Reporting of clinical trial research varies and is known to be poor.

**What are the new findings**

- The REPORT Trial guide is a one-stop, ‘how-to’ implementation guide and directory that holds tacit knowledge and references to first-choice sources of information about effective and transparent trial reporting (eg, CONSORT).
